# Differential Expression of Collectins in Human Placenta and Role in Inflammation during Spontaneous Labor

**DOI:** 10.1371/journal.pone.0108815

**Published:** 2014-10-10

**Authors:** Ajit Kumar Yadav, Hemangi Chaudhari, Himangi Warke, Premanand Keshavlal Shah, Eswari Dodagatta-Marri, Uday Kishore, Taruna Madan

**Affiliations:** 1 Department of Innate Immunity, National Institute for Research in Reproductive Health (Indian Council of Medical Research), Mumbai, Maharashtra, India; 2 Department of Obstetrics and Gynecology, Seth Gordhandas Sunderdas Medical College and King Edward Medical (KEM) Hospital, Mumbai, Maharashtra, India; 3 Centre for Infection, Immunity and Disease Mechanisms, College of Health and Life Sciences, Brunel University London, Uxbridge, United Kingdom; Oxford University, United Kingdom

## Abstract

Collectins, collagen-containing Ca^2+^ dependent C-type lectins and a class of secretory proteins including SP-A, SP-D and MBL, are integral to immunomodulation and innate immune defense. In the present study, we aimed to investigate their placental transcript synthesis, labor associated differential expression and localization at feto-maternal interface, and their functional implication in spontaneous labor. The study involved using feto-maternal interface (placental/decidual tissues) from two groups of healthy pregnant women at term (≥37 weeks of gestation), undergoing either elective C-section with no labor (‘NLc’ group, n = 5), or normal vaginal delivery with spontaneous labor (‘SLv’ group, n = 5). The immune function of SP-D, on term placental explants, was analyzed for cytokine profile using multiplexed cytokine array. SP-A, SP-D and MBL transcripts were observed in the term placenta. The ‘SLv’ group showed significant up-regulation of SP-D (p = 0.001), and down-regulation of SP-A (p = 0.005), transcripts and protein compared to the ‘NLc’ group. Significant increase in 43 kDa and 50 kDa SP-D forms in placental and decidual tissues was associated with the spontaneous labor (p<0.05). In addition, the MMP-9-cleaved form of SP-D (25 kDa) was significantly higher in the placentae of ‘SLv’ group compared to the ‘NLc’ group (p = 0.002). Labor associated cytokines IL-1α, IL-1β, IL-6, IL-8, IL-10, TNF-α and MCP-1 showed significant increase (p<0.05) in a dose dependent manner in the placental explants treated with nSP-D and rhSP-D. In conclusion, the study emphasizes that SP-A and SP-D proteins associate with the spontaneous labor and SP-D plausibly contributes to the pro-inflammatory immune milieu of feto-maternal tissues.

## Introduction

Immunological components are an essential part of mammalian pregnancy due to semi-allogenicity of the fetus. Fetal tolerance is considered to be induced via an immunological cross-talk by the spatial and temporal expression of pro-inflammatory and anti-inflammatory molecules in the gestational tissues [1], [2]. At term, a functional transition in these tissues occurs from anti-inflammatory to pro-inflammatory leading to parturition. The pro-inflammatory immune signature includes matrix metalloproteinases (MMPs; MMP-2 and 9), and pro-inflammatory cytokines such as IL-1β, IL-6, IL-8 and TNF-α [3], [6]. Physiological events in parturition involve rupture of the membranes, cervical ripening and dilatation, contractility of the myometrium, decidual reaction, placental separation and uterine involution [3], [4], [5], [6], [7], [8]. However, the nature of the immune receptors involved in this functional switch is not fully characterized [8]. Further, the functions of glycosylated immune receptors are modulated by specific lectins [9]. For instance, glycodelins and galectins play a major role in modulating the fetal-reacting maternal immune cells at the feto-maternal interface through gestation [10], [11].

Collectins, collagen-containing Ca^2+^ dependent C-type lectins, are secretory proteins integral to immunomodulation and host defense. Members of this family include surfactant protein A (SP-A), surfactant protein D (SP-D) and mannan binding lectin (MBL), which share the human chromosomal location between 10q11.2–10q23.1 [12]. SP-A and SP-D are synthesized and secreted by type II lung alveolar cells and non-ciliated bronchiolar epithelial cells, whereas hepatocytes secrete serum MBL [12], [13]. SP-A and SP-D actively participate in phagocytosis, antigen presentation, oxidative burst, apoptotic cell clearance and T cell immunomodulation [12]. Mostly, orientation of SP-A and SP-D during interaction with the receptors on immune cells brings about either a pro-inflammatory (via calreticulin-CD91 complex) or anti-inflammatory (via SIRP-α) immune response [14]. Serum MBL initiates the lectin complement pathway and modulates the immune response through TLRs, in developing the humoral immune response [13].

At extra-pulmonary locations, synthesis of SP-A, SP-D and MBL, has been reported in the human lower female reproductive tract and these proteins are implicated in protection against reproductive tract infections [15], [16], [17]. SP-D is primarily localized to the luminal and glandular epithelium of the human uterus in the secretory and menstrual phases [18]. SP-A (SP-A1 and SP-A2) and SP-D transcripts have been observed in the term amnion and chorio-decidual tissues [19]. Levels of SP-A and MBL increase in the cervico-vaginal lavage during early follicular phase and secretory phase, respectively [15], [16]. SP-A and SP-D have been localized in the 4^th^ to 8^th^ week gestational villous and extra villous trophoblast [20], and in term human placenta [21]. SP-A synthesis in term chorio-amniotic membrane increases in chorioamnionitis but not in spontaneous labor [22], and could be induced by cortisol in the chorionic trophoblast [23]. A significant increase in the maternal serum MBL commencing at 12 weeks of normal pregnancy and decrease at post-partum has been observed [24]. Extra-hepatic MBL synthesis occurs in intestine and testis but has not been reported in human gestational tissues [25].

A progressive increase in the levels of SP-A, SP-D and MBL proteins has been reported in human amniotic fluid (AF) with advancing gestation [26]. A decrease in the levels of SP-A in AF was associated with the spontaneous labor but not with intra-amniotic infection [26], [27]. Previously, the intra-amniotic injection of human SP-A to pregnant female mice led to a preterm delivery while, administration of polyclonal antibody to SP-A resulted in post-term delivery [28]. In human, redistribution of AF SP-A occurs between amnion and amniotic fluid during labor and protects amnion through an anti-inflammatory function [29].

Conspicuously, AF has been anticipated as the major source for SP-A, SP-D and MBL proteins in placenta. Thus, we investigated placental synthesis, labor associated differential expression and localization of the collectins at feto-maternal interface, and their functional implication in spontaneous labor.

## Materials and Methods

### Study participants

Term feto-maternal tissues were obtained from two groups of participants, five from women undergoing Caesarean section with no labor (‘NLc’ group, n = 5) and five from women undergoing normal vaginal delivery with spontaneous labor (‘SLv’ group, n = 5) at the Department of Obstetrics and Gynaecology, Seth G.S. Medical College and KEM Hospital, Parel, Mumbai, with written informed consent obtained prior to the sample collection. Comparison of these two study groups involves one more variable (mode of delivery), besides onset of labor, and different modes of delivery may affect placental mRNA and protein expression profiles. The study design excluded the participants undergoing normal labor with caesarean section owing to association with more variables. The study was approved by the Institutional Ethics Committee for Clinical Research (152/2009-NIRRH), NIRRH (ICMR) and Ethics Committee for Research on Human Subjects (EC/GOVT-7/2009), Seth G.S. Medical College and KEM Hospital, Parel, Mumbai. All the women were in the normal fertility period (20–40 years), and had singleton pregnancy (>37 week of gestation; average 38.5±0.5), with normal child birth weight. Medically, no reproductive tract infections, chronic diseases, or pathophysiological conditions of pregnancy were observed or reported by the participants and they did not receive any hormone therapy. The gestation matched feto-maternal tissue samples were collected in sterile PBS/saline in both groups.

The collected placental cotyledon was dissected under stereomicroscope for the feto-maternal tissues. The separated placental and decidua basalis tissues were used for further analysis in the study. Hematoxylin and Eosin staining was performed to examine the morphology of decidual and chorionic villi, and cytokeratin 7 staining was used to distinguish the isolated trophoblasts from decidual tissues in both the study groups (data not shown). The placental or decidual samples were partly (a) suspended in 10% neutral formaldehyde (NBF) for immunohistochemical analysis, (b) stored at −80°C in trizol for real time RT-PCR analysis, and (c) stored at −80°Co in lysis buffer for Western blot analysis.

### Purification of human SP-D (nSP-D)

Human term amniotic fluid was collected and pooled from women at term undergoing C-section (n = 20) at the Department of Obstetrics and Gynaecology, Seth G.S. Medical College and KEM Hospital, Parel, Mumbai, with written informed consent from the participants obtained prior to the sample collection. The pooled amniotic fluid was stored at −20°C until further use for protein purification. The study was approved by the Institutional Ethics Committee for Clinical Research (153/2009-NIRRH), NIRRH (ICMR) and Ethics Committee for Research on Human Subjects (EC/GOVT-6/2009-KEM), Department of Obstetrics and Gynaecology, Seth G.S. Medical College and KEM Hospital, Mumbai.

The SP-D protein was purified with modifications in the protocol reported earlier using reagents sourced from Sigma, USA [30]. Briefly, the pooled AF was thawed and made to a 5 mM final concentration with EDTA and incubated for 30 min prior to centrifugation at 11,000 rpm for 30 min. Supernatant was adjusted to 20 mM Tris-HCl, 150 mM NaCl and 30 mM CaCl_2_ and pH 7.4. Pre-equilibrated maltosyl-agarose beads with TCB (20 mM TrisHCl, 10 mM CaCl_2_ and 0.02% sodium azide (w/v), were added to supernatant in 20 ml/L ratio and the pH was readjusted to 7.4. The SP-D and maltose-agarose binding was carried out at room temperature for 2 h with mild shaking. Maltosyl-agarose beads were washed extensively with TCB, and 1 M and 0.5 M NaCl in TCB to remove any non-specific interactions. Elution of SP-D proteins was performed by 150 mM MnCl_2_ (in 20 mM Tris pH 7.4) at room temperature for 2 h or overnight at 4°C on shaker. The tubes were centrifuged and supernatant containing purified SP-D was collected. Rest of the SP-D strongly associated with the beads was eluted with 20 mM EDTA in 20 mM Tris-HCl, pH 7.4 at room temperature with mild shaking for 1 h. Supernatant collected after MnCl_2_ elution contained SP-D proteins only, while EDTA eluted fractions contained both SP-A and SP-D proteins and they were further separated by gel filtration and analyzed by silver staining (**Fig.S3.i**) and Western blot respectively (**Fig S3.ii**). The purified nSP-D protein was assayed for endotoxin content (given below), its binding and ability to agglutinate *A. fumigatus* conidia and its potential to enhance killing of fungal conidia by human PBMCs *in vitro* [31].

### Purified recombinant fragment of human SP-D (rhSP-D)

The recombinant fragment of human SP-D containing neck and CRD domains and a part of collagen domain (rhSP-D) was expressed under the bacteriophage T7 promoter as inclusion bodies in *Escherichia coli* as described earlier [30], [31]. Cross-linking studies indicated that the rhSP-D existed predominantly as a trimer in the solution. Its identity was confirmed by N-terminal sequencing, and was judged to be pure by SDS-PAGE and immunoblotting [Fig S3 (i and ii)]. The rhSP-D preparation and purified SP-D were examined for endotoxin levels using the QCL-1000 Limulus amebocyte lysate system (BioWhittaker Inc., Walkersville, Maryland, USA). The assay was linear over a range of 0.1–1.0 EU/ml (10 EU  = 1 ng of endotoxin) and the amount of endotoxin present in the preparations was estimated to be 4 pg/µg of rhSP-D and 2 pg/µg of purified nSP-D.

### Real-time RT-PCR analysis of transcripts

The placental total RNA was isolated by the trizol method and 3 µg was reverse transcribed using Superscript III first strand synthesis kit (Invitrogen, USA). 1–3 µl of the cDNA was used for each 25 µl real time PCR reaction using SYBR green (Biorad, USA). Primers were custom-made by Sigma Oligos (Genex, India). Details about the primer pair sequences, annealing temperatures and product size is given in the Table 1. The transcripts of the SP-A were analyzed using two primer pairs, in view of the earlier report showing predominant presence of SFTPA-1 (SP-A1) in chorioamniotic membrane and trophoblasts [19]. First primer pair could amplify SFTPA-2 (SP-A2), and the second primer pair could amplify both SFTPA1 (SP-A1) and SFTPA2 (SP-A2), including their transcript variants.

**Table 1:** Details of primer pair sequences, annealing temperature and product length for amplification of each transcript.

Transcripts	Forward Primer (5′->3′)	Reverse Primer (5′->3′)	Annealing Temperature (°C)	Product length
SP-A	TTTATAAGGACACTGAAGCT	TCATCTTTATTCAGCTCAGG	53	145
SP-A2	CAGTACCGGCCAAGCATAAT	CATGTCAGTCACAGGGTTGG	58.8	194
SP-D	AGGCTGCTTTCCTGAGCATGAC	CCATTGGTGAAGATCTCCACACAG	57.8	148
MBL	GCCACCCCCAGGAATGCTG	GAGCAGGGGACGTCATTCCA	59	224
18S	GGAGAGGGAGCCTGAGAAAC	CCTCCAATGGATCCTCGTTA	62	171

Real time RT-PCR was carried out with a hot start at 95°C for 3 min and amplified at 95°C–30 sec; 56°C–30 sec; 72°C–30 sec; for 40 cycles and final extension at 72°C for seven minutes on Biorad-iCycler (Biorad, USA). The semi-quantitation of the transcripts was carried out using ΔΔC_t_ standard method after normalizing with the 18S rRNA abundance in the same samples. Melting curve analysis was conducted to determine the specificity of the amplified products and to ensure the absence of primer-dimer formation. All the products yielded the predicted melting temperature and product size on the gel. All the analyses were performed in triplicates and the mean value was taken for further calculations.

### Localization of Collectins at feto-maternal interface

Serial placental or decidual tissue sections of 5 µm thickness were collected on the poly-L-lysine coated slides. Sections were deparafinized in xylene and gradually rehydrated with 90%, 70%, 50%, 30% and distilled water each for 5 min. Endogenous peroxidase activity was blocked by 3% H_2_O_2_ in methanol, and antigens were retrieved by boiling the slides in sodium citrate buffer (H3300, Vector, USA). The tissue sections were blocked in 5% normal goat serum (NS1, GeNei, India) for 1 h at room temperature in antibody diluents (S2022, Dako, CA, USA) and probed overnight with mouse monoclonal antibody to SP-A (ab49566, Abcam, MA, USA) (1: 20), polyclonal SP-A antibody raised in goat (ab7245, Abcam, USA) (1: 100), mouse monoclonal SP-D antibody (ab15688, Abcam, USA) (1: 50), or mouse monoclonal antibody to MBL (ab23457, Abcam, USA) (1: 50) in antibody diluent at 4°C. The kits for immunohistochemistry (K5003, Dako, USA) were applied further as prescribed by the manufacturer. Tissue sections were incubated with biotinylated secondary antibody reagent for 2 h and signals were enhanced using avidin conjugated HP for 30 min at room temperature. For polyclonal goat antibody to SP-A, secondary anti goat IgG raised in donkey, conjugated with HRP (sc2033, Santacruz, TX, USA) (1: 250) was used for 3 h in moist chamber at room temperature. The sections were incubated with 3-amino-9-ethylcarbazole (AEC) substrate for 10 min in dark and counter stained with hematoxylin (Dako, USA) for 3-5 minutes. For the negative controls, tissue sections were treated with antibody diluent alone instead of the primary antibody. Slides were mounted using D.P.X mountant (Qualigens, India) and observed under the microscope. All the samples for each individual antibody were processed using the same protocol and at the same time. Photomicrographs were taken with an Axioplan microscope (Zeiss, Oberkochen, Germany). The immunoperoxidase staining was quantified using NIS Elements Imaging Software ver 4.10 (Nikon Corporation, Tokyo, Japan). Expression levels of collectins were calculated by multiplication of the positive area and mean optical intensity [32].

### Western blot analysis of collectins in placental and decidual tissues

The placental or decidual (∼100 mg) tissue was minced and homogenized in 0.5 ml of lysis buffer containing 8 M urea (Sigma) and 2% CHAPS (GE Healthcare, USA) supplemented with protease inhibitor cocktail and nucleases (GE Healthcare, USA). Samples were vortexed for 1 h followed by centrifugation at 13,000 rpm for 40 min at 4°C. Protein concentration in the supernatants was estimated using the Micro BCA kit (Thermoscientific, USA). A total of 30 µg placental protein was loaded in each lane and resolved on 10% SDS-PAGE, using reagents obtained from Sigma unless mentioned. Proteins were transferred onto nitrocellulose membrane (HATF00010, Millipore, USA) and membranes were blocked in 5% non-fat dry milk in TBS overnight at 4°C. The excess of milk protein was removed by washing the blot with washing buffer (TBS-0.002% TritonX-100). Blot was probed with mouse monoclonal antibody to SP-A (ab49566, Abcam, USA) (1: 150), mouse monoclonal antibody to SP-D (ab15688, Abcam, USA) (1∶150), mouse monoclonal antibody to MBL (ab23457, Abcam, USA) (1∶250), and polyclonal anti SP-A raised in goat (ab7245, Abcam, USA), (1∶1000), in TBS containing 1% nonfat dry milk. The polyclonal β-actin antibody raised in rabbit (ab8227, Abcam, USA) (1∶2000) was used for normalization. Polyclonal anti-mouse IgG raised in goat conjugated with HRP (P0447, Dako, USA) (1: 1000) or anti-rabbit IgG antibody raised in goat linked with HRP (P0448, Dako, USA) (1: 1000) or anti-goat IgG raised in donkey conjugated with HRP (sc2033, Santacruz, USA) (1: 5000) in TBS containing 1% nonfat dry milk were used and signals were detected using femto-level chemi-luminiscence detection method (Thermoscientific, USA). In view of the presence of several activated proteases, including MMP9, in term placenta undergoing labor and ability of MMP9 to proteolytically cleave SP-D, we evaluated the MMP9 cleaved SP-D product in the term placenta of the two groups using monoclonal antibody to SP-D (ab15688, Abcam, USA) (1∶150) [33]. Protein bands corresponding to SP-A, SP-D, cleaved SP-D and MBL were quantified using β-actin as reference control using Labscan and Image Quant TL (GE Healthcare, USA) software based on densitometry. The experiments were carried out three times for each protein. The quantitative data is presented as mean ±SEM.

### Effect of SP-D on cytokine secretion by placental explants

The placental tissue explants (∼100 mg) from C-section (‘NLc’ group) were prepared within half an hour of surgery and cultured in the DMEM/F12 (D8900, Sigma, USA) and supplemented with 1% antibiotics and 10% FBS (Gibco, USA) in presence or absence of either purified human SP-D (nSP-D) or purified recombinant fragment of human SP-D (rhSP-D) dissolved in PBS. The placental explants (n = 3) in triplicate were treated with PBS (control) or 0.5, 1.0 and 5 µg/ml of nSP-D/rhSP-D in a 24 well culture plate. The culture plates were incubated at 5% CO_2_, 37°C for six hours. The culture medium and explants were recovered for further analysis of cytokines and transcripts. The levels of cytokines secreted were evaluated using the multiplex cytokine array comprising of twelve cytokines (EV3623, Randox Laboratories, UK). The mean value of each cytokine was obtained for every sample and presented as mean ±SEM.

### Placental explant viability assay

The MTT [3-(4,5-dimethylthiazol-2-yl)-2,5 diphenyltetrazolium bromide; Calbiochem] assay was used to evaluate the effect of nSP-D/rhSP-D proteins on the viability of placental explants. The culture medium of treated or untreated placental explants was removed after 6 h incubation, and the explants were washed with sterile PBS. The placental explants were transferred to the 0.25 mg/ml MTT solution in sterile PBS and incubated for 2 h. The freeze-killed placental tissue was used as negative control in the experiments. At the end of incubation, the MTT solution was drained and acidified isopropanol (0.02N HCl) was added to extract the formazan produced by viable cells in the tissue explants. Absorbance of extracted formazan was measured at 540 nm, normalized with absorbance of the freeze-killed explants and plotted as percentage of the control [Fig.S4(c)]. The experiment was performed in triplicate for each sample.

### Statistics

The relative quantitation was evaluated by one way ANOVA using MS Excel or GraphPad, and significance was set at p<0.05.

## Results

### Collectins are synthesized in placental tissues

The transcript analysis showed the synthesis of SP-A (both SP-A1 and SP-A2), SP-D and MBL in the human term placenta (**Fig. S1**). The immunoblot analysis further confirmed the presence of SP-A, SP-D and MBL proteins in the term placenta. Interestingly, the monoclonal antibody to SP-A, identified two major protein bands at ∼38 kDa and ∼46 kDa in the placenta, besides a ∼34 kDa minor protein band (**Fig.S2a**), while the human term amniotic fluid showed two bands of SP-A at 28–34 kDa and ∼50 kDa. The monoclonal antibody to SP-D identified two reported [49] SP-D protein bands of monomer 43 kDa and the glycosylated form of 50 kDa in the amniotic fluid and placenta (**Fig.S2b**). The monomeric form of serum MBL protein of 32 kDa, was present in the human term amniotic fluid and placenta (**Fig. S2c**). The synthesis of SP-A, SP-D and MBL and presence of proteins in term placenta prompted us to investigate their expression during the spontaneous labor.

### Differential expression of SP-A and SP-D

**Figure 1:**
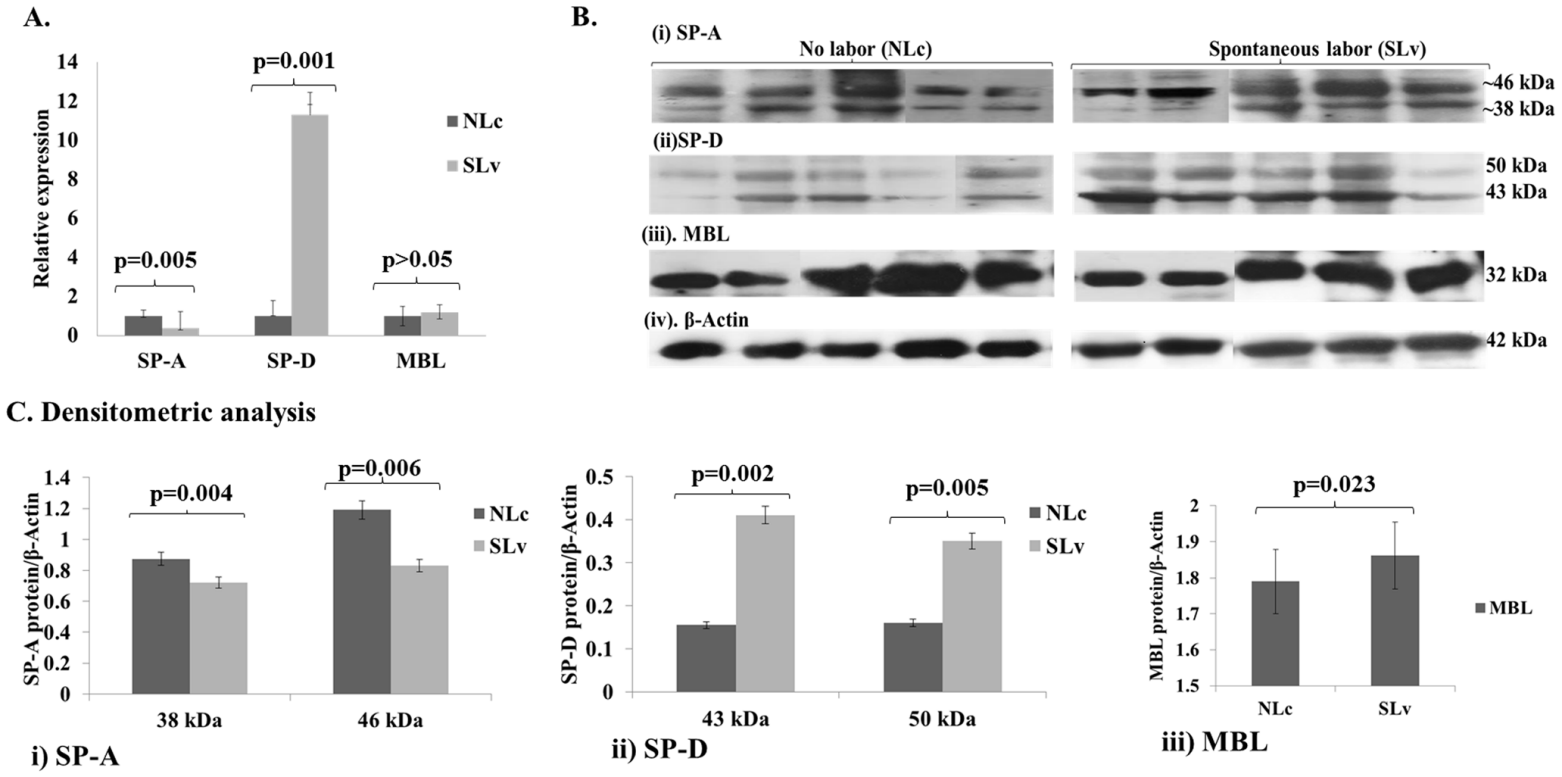
Differential expression of SP-A, SP-D and MBL in term placenta (n = 5/group). A) Semi-quantitation of the transcripts assessed by real time RT-PCR between two groups of placenta from caesarean section with no labor (‘NLc’) and placenta from normal vaginal delivery with spontaneous labor (‘SLv’). The experiments were carried out 3 times for each transcript and relative quantity was calculated by 2^−ΔΔCt^ method after normalizing with 18S rRNA of the respective sample. The relative fold expression presented is compared to controls; error bars represent ±SEM for each transcript. B) Western blot analysis of SP-A, SP-D and MBL in total placental protein (30 µg) of both groups (‘NLc’ vs ‘SLv’; n = 5/group). The blots were probed with mouse monoclonal antibody to SP-A (i), SP-D (ii), MBL (iii), and rabbit polyclonal antibody to β-actin for normalization (iv). C) Densitometric analysis of blots for SP-A, SP-D and MBL proteins using Labscan and ImageQuant TL analysis tools (GE Healthcare, USA) and presented as mean ±SEM; i) SP-A, ii) SP-D and iii) MBL. Statistical analysis was performed with one way ANOVA at p<0.05.

The differential expression analysis of the SP-A, SP-D and MBL transcripts was carried out in term placental tissues of the spontaneous labor group (‘SLv’) and placenta from C-sectionno labor (‘NLc’) groups. Transcript analysis revealed a significant up-regulation of the SP-D (∼11 fold, p = 0.001) in the placental tissues of ‘SLv’ group compared to the ‘NLc’ group (**Fig.1A**). The same ‘SLv’ group placental tissues showed significant association with decreased levels of SP-A transcripts (p = 0.005), compared to the ‘NLc’ group. In addition, use of SP-A primers for both SP-A1 and SP-A2 transcripts did not result in any difference in the relative expression compared to the primers for SP-A2 transcript (data not shown). In contrast, a slight increase in the MBL transcript (**p>0.05**), was observed in the placental tissues of ‘SLv’ group compared to the ‘NLc’ group [**Fig.1A**].

The quantitative analyses of SP-A, SP-D and MBL proteins were carried out in placental tissues of both the groups. Similar to the transcript expression data, we found a significant decrease in ∼38 kDa and ∼46 kDa protein bands of SP-A by 2 fold (p = 0.004) and 1.4 fold (p = 0.006) respectively in the placenta of ‘SLv’ group compared to the ‘NLc’ group [**Fig.1B (i) and C (i)**]. With polyclonal goat anti-SP-A antibody (ab7245), that is reported [29] to identify 68 kDa and ∼75 kDa SP-A oligomers, a decrease in the SP-A was confirmed in the ‘SLv’ group compared to the ‘NLc’ group (data not shown). In contrast, up-regulation in the levels of SP-D was observed for the monomeric 43 kDa and differentially glycosylated 50 kDa forms. However, SP-D forms of ∼43 kDa and ∼50 kDa showed only 2.6 fold (p = 0.002) and 2.2 fold (p = 0.005) increase respectively in the spontaneous labor group, a considerably lesser increase than the transcript levels [**Fig. 1B (ii) and C (ii)**]. Increase in the MBL protein of ‘SLv’ group was not found to be significant (p>0.05) when compared with ‘NLc’ group [**Fig. 1B (iii) and C (iii)**].

**Figure 2:**
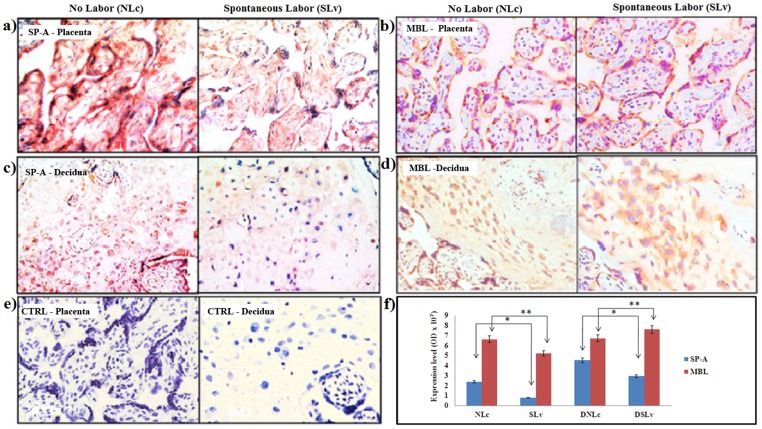
Immunolocalization of SP-A and MBL proteins at feto-maternal interface (n = 5/group). The 5 µm thin tissue sections a) placenta or c) decidua were probed with monoclonal antibody to SP-A. Anti-MBL monoclonal antibody was applied to b) placenta or d) decidua. e) Negative controls for the respective tissues. The tissue sections were counterstained with hematoxylin and mounted with DPX. Representative images are 400× original magnification. f) The relative intensity of SP-A and MBL staining was quantitated by NIS elements version 4.1 software analysis tools. Bars are mean ±SEM, *p<0.05 and **p>0.05.

Immunohistochemistry was used to evaluate the localization of SP-A, SP-D and MBL proteins in the placental and decidual tissues of both groups. The mesenchymal stromal cells of the chorionic villi from both groups were intensely stained for SP-A protein (**Fig. 2a**). In addition, chorionic villi from the ‘NLc’ group showed more intense SP-A staining in the syncytiotrophoblast layer compared to the ‘SLv’ group. The MBL protein mostly localized in the syncytiotrophoblast layers with occasional staining in stroma of both the groups (**Fig. 2b**).

Similarly, cellular and matrix staining of SP-A and MBL was observed in decidua of ‘SLv’ group (‘DSLv’) compared to ’ ‘NLc’ group (‘DNLc’) [**Fig. 2c and d**]. The expression levels confirmed significant decrease in the SP-A proteins in placental (**p<0.05**) and decidual tissues (**p<0.05**) of the ‘SLv’ group compared to the ‘NLc’ group (Fig.2f). Similar to the transcript and protein data, the expression levels of MBL proteins were not significantly different in the two groups (**Fig. 2f**).

### SP-D is increased in syncytiotrophoblast and decidual tissue of labor

**Figure 3:**
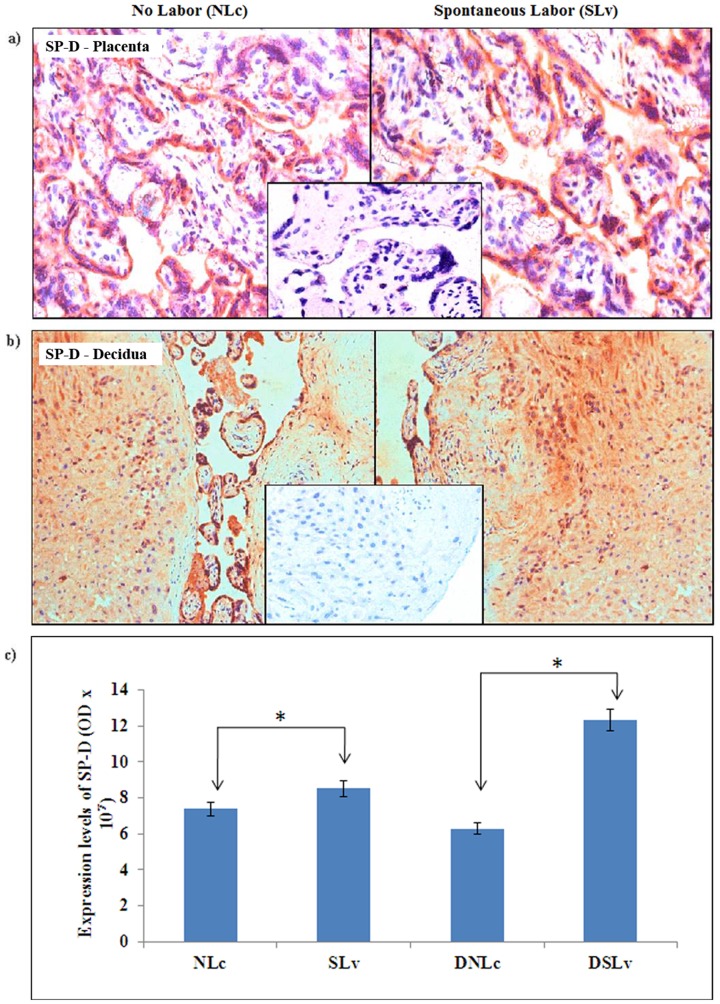
Immunolocalization of SP-D at the term feto-maternal interface (n = 5/group). The 5 µm thin tissue sections were probed with monoclonal antibody to SP-D a) placenta (‘NLc’ vs. ‘SLv’); b) decidua (‘DNLc’ vs. ‘DSLv’) and subsequently counter stained with hematoxylin. Representative images are 400× original magnification (a) or 200× original magnification (b), insets are negative controls for the respective tissues. c) The relative intensity of SP-D staining in two groups of placenta and decidua was quantitated by NIS elements version 4.1 software analysis tools. Bars are mean ±SEM; *p<0.05.

**Figure 4:**
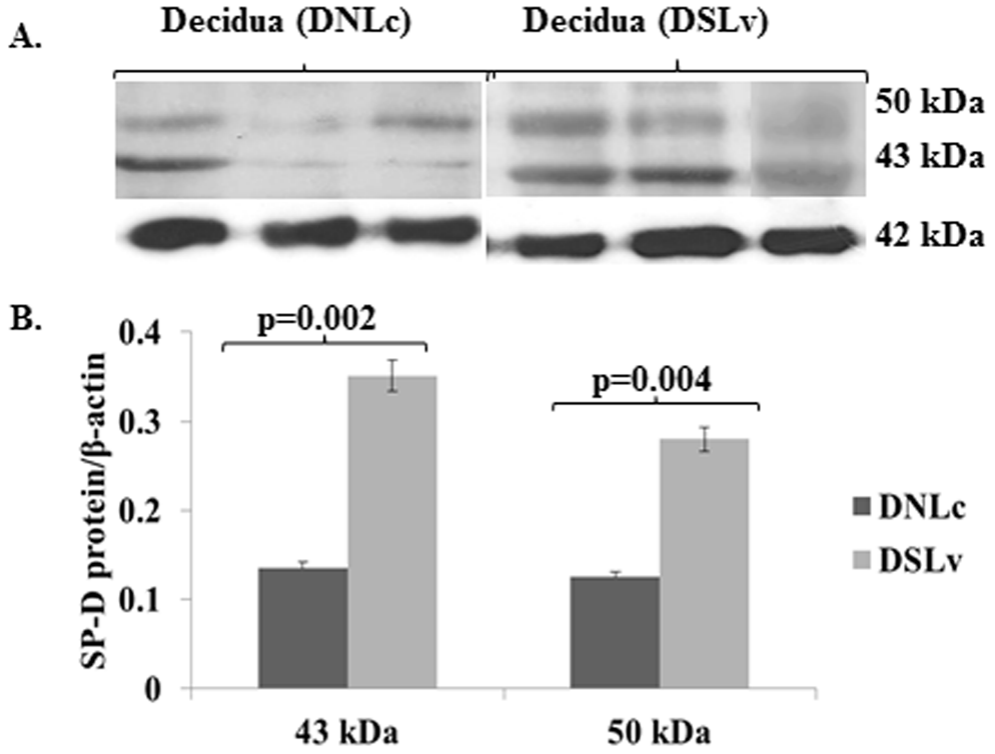
Western blot analysis of SP-D in term decidual tissues (‘DNLc’ vs. ‘DSLv’). Total term decidual proteins (30 µg each sample) were blotted and probed with mouse monoclonal antibody to SP-D or polyclonal antibody to β-actin; A) representative images (n = 5/group) B) Relative quantity of SP-D in each group was obtained by Labscan and 1D ImageQuant analysis tools using β-actin as internal reference control for normalization. Bars are mean ±SEM; *p<0.05.

Although SP-D is localized in the glandular epithelium of the secretory phase human endometrium, it has not been reported in the term decidua during labor. To dissect the relevance of the SP-D protein in placental- decidual cross talk, localization and level of SP-D protein were analyzed (Fig. 3a and 3b). The SP-D protein was observed in syncytiotrophoblast and mesenchymal stromal cells, and matrix of mesenchymal stromal cells of both the groups (**Fig. 3a**). Intriguingly, we observed intense staining for SP-D in the mesenchymal stromal cells compared to the syncytiotrophoblast layers of the villi in the ‘NLc’ group. The quantitative analysis revealed a significant increase in the SP-D protein expression levels in the placenta (**p<0.05**) and decidua (**p<0.05**) of ‘SLv’ group compared to the ‘NLc’ group **(** **Fig.3a, 3b and 3c** **)**. In the Western blot analysis of decidua, similar to the placenta, significant increase in both the isomers of SP-D protein was observed in the ‘SLv’ group, with more prominent increase in 43 kDa (**p = 0.002**) than 50 kDa isomer (**p = 0.004**) (**Fig. 4A and B**). Together, these data indicated that SP-D proteins increase in the syncytiotrophoblast cell layers of placenta during labor. We observed a significant association of SP-A, SP-D and MBL proteins with the syncytial knots in both the groups (data not shown).

### MMP9 cleaved SP-D is increased in placental tissue of labor

Matrix metalloproteinases, involved in collagenolysis, are the key molecules up-regulated during labor. SP-D is known to be cleaved by MMP9 [33], resulting in a 25 kDa fragment containing CRD (**Fig. S4a**). We asked if the levels of SP-D are altered in placental tissues due to MMPs during spontaneous labor. The 25 kDa SP-D fragment was increased in the placental tissues of the ‘SLv’ group compared to the ‘NLc’ group (**Fig. S4b**).

### SP-D augmented the pro-inflammatory cytokine secretion

**Figure 5:**
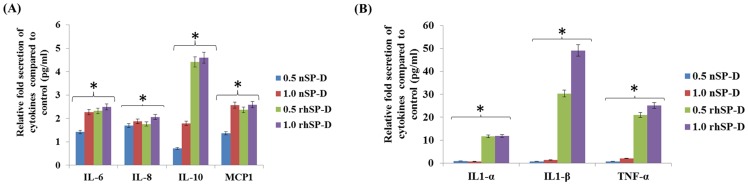
Effect of rhSP-D and nSP-D on cytokine secretion by term placental explants. The term placental explants (C-section, n = 3) were treated with media containing different concentrations of nSP-D or rhSP-D and were compared with media treated control. The secreted cytokines in the media supernatant were analyzed using a biochip for twelve cytokines (Randox Laboratories, UK). A) Relative fold change in IL-1α, IL-1β and TNF-α cytokines for each concentration of nSP-D and rhSP-D compared to control. B) Relative fold change in IL-6, IL-8, IL-10 and MCP-1 cytokines for each concentration of nSP-D and rhSP-D compared to control. Bars represent mean ±SEM; *p<0.05.

To get an insight into the relevance of increased SP-D levels in the placenta, the *in vitro* immune effects of nSP-D or rhSP-D on placental explants of the ‘NLc’ group were evaluated. The placental explants were treated with 0.5, 1.0 and 5.0 µg/ml concentration of either nSP-D or rhSP-D and PBS treated explants were used as controls. Both nSP-D and rhSP-D induced the pro-inflammatory cytokine secretion by placental explants in a dose dependent manner (**Fig. 5A and B**) (data for 5.0 µg/ml concentration not shown). We found a significant increase in the pro-inflammatory cytokine secretion with 1.0 µg/ml concentration of either nSP-D or rhSP-D in comparison to the controls. While the secretion of IL-2 and IL-4 cytokines was not significantly affected with 1.0 µg/ml of rhSP-D, levels of IL-1α, IL-1β, and TNF-α increased significantly (p<0.05) (**Fig. 5A**). Levels of IL-6, IL-10 and MCP-1 were also significantly increased with 1.0 µg/ml of nSP-D and rhSP-D (p<0.05), compared to the untreated placental explants (**Fig. 5B**). There were no significant alterations in the levels of VEGF, EGF and IFN-γ (p>0.05). Viability of the nSP-D or rhSP-D treated placental explants was not significantly different from that of the PBS treated controls (**Fig. S4c**).

## Discussion

We report the synthesis of transcripts of three members of the collectin family: SP-A, SP-D and MBL, by the human term placenta and their expression and localization at the feto-maternal interface during spontaneous labor. Comparative analysis of the placental and decidual tissues from the study participants of two groups ‘spontaneous labor’ (‘SLv’ group) and ‘no labor’ (‘NLc’ group) suggested significant up-regulation of expression of SP-D and down-regulation of SP-A during spontaneous labor.

This study, for the first time, showed association of a significant increase in the SP-D at the feto-maternal interface with spontaneous labor. An important role of SP-D in the parturition process was evident by significant association of the Met31Th SP-D gene polymorphism in the fetus, but not in mothers, with the spontaneous preterm birth in Finnish population [34]. The threonine allele increases the concentration of lower oligomeric forms with differential binding affinity for ligands compared to higher order SP-D oligomers [35]. However, polymorphisms in embryonic or maternal SP-A and MBL alleles did not show any significant association with the spontaneous preterm birth in the Finnish population [34]. In addition, mice overexpressing rat SP-D showed enhanced pro-inflammatory cytokine production in the fetal and maternal compartments upon maternal LPS exposure [36]. SP-A^−/−^, SP-D^−/−^ and SP-A/D^−/−^ mice delivered at term during the first pregnancy. However, a significant delay in the parturition was observed during second pregnancy in SP-A/D^−/−^ mice and was attributed to decrease in production of IL-1α and IL-6 in the myometrial tissue [37].

The labor associated decrease of SP-A in the decidual tissues is in coherence with a recent report that SP-A was highly expressed in the decidual (vimentin-positive) cells from tissues collected before labor and was significantly reduced in the tissues collected after labor [38]. However, in contrast to the study by Snegovskikh *et al*., we observed the presence of SP-A transcript as well as protein in the term trophoblasts of both study groups [38]. Intra uterine administration of SP-A has been shown to enhance the expression of IL-10 and CXCL1 and prevent preterm delivery in mouse, while human placental amnion treated with SP-A decreased the IL-1β secretion, indicating an anti-inflammatory function of SP-A [39], [29]. It is plausible that feto-maternal SP-A prevents an inflammatory response till the term and to support the spontaneous labor that is predominantly an inflammatory process, placental synthesis of SP-A is significantly down-regulated.

Expression of the three collectins is differentially regulated by different transcription factors even though they are located in close vicinity of each other (human chromosome region (10q11.2–10q23.1), and have sequential similarity. The TTF-1 (thyroid transcription factor-1) is essential for expression of SP-A in the lung cells [40]. Expression of TTF-1 is induced by glucocorticoids, cAMP and TGF-β, while pro-inflammatory cytokine TNF-α inhibits its expression [40]. Cortisol induced SP-A mRNA in trophoblasts at lower concentration (0.3×10^−6^ M), but inhibited at the higher concentration (10^−5^M) [23]. Expression of SP-D is regulated by several transcription factors such as, AP-1, TTF-1, nuclear factor of activated T cells (NFAT) and HNF-3 [41] while expression of MBL is regulated by HNF-3 [42].

Oxidative stress, an important factor during pregnancy, could affect the expression of anti-oxidant SP-A and SP-D molecules at the feto-maternal interface [43], [44]. Interestingly, oxidant H_2_O_2_ decreases SP-A, and increases SP-D transcripts via MAPK/JAK-STAT pathway in human lung cells. Glucocorticoids show a similar effect through specificity protein-1 (SP-1) in fetal lung explants culture [45], [46].

Further, this paradoxical regulation of SP-A and SP-D expression could be due to the decreased stability of SP-A mRNA in presence of higher concentrations of glucocorticoids [47]. Interestingly, SP-A^−/−^ mice show seven fold elevated levels of SP-D, suggesting that the two genes regulate each other via feedback mechanisms [48].

Various forms of SP-A and SP-D protein were observed in the present study (two major SP-A protein bands of ∼38 kDa and ∼46 kDa in the placental tissues and 28–34 kDa and 50 kDa isomers of SP-A in term amniotic fluid) as reported previously [49], [50]. The 2D analysis revealed that the isoelectric points of various SP-A protein forms isolated from the lung range between pI 4.5–5.4 and their molecular weights range from 20.5–26 kDa (five forms), 26–32 kDa (eight forms), and 32–42 kDa (three forms) [49]. Monoclonal antibody to human SP-D identified 43 kDa and its 50 kDa glycosylated form in human term amniotic fluid, placental and decidual tissue extracts as reported earlier [50]. We did not observe any tissue specific MBL forms in the human placental samples analyzed. The placental synthesis of the MBL may contribute to the increased levels of MBL observed in maternal serum and mediate the interaction of extra villous trophoblast and decidual endothelial cells during normal pregnancy [24], [51].

Increase in pro-inflammatory immune response in gestational tissues during normal delivery contributes to cervical tissue ripening and decidual reaction leading to labor [3], [4], [5], [6], [7], [8]. In the present study, both nSP-D and rhSP-D induced a dose dependent pro-inflammatory immune response in the term placental explants suggesting involvement of the CRD domain of SP-D. A higher increase in the cytokines inducedby rhSP-D than nSP-D could be due to the difference in molar concentrations of nSP-D (1 µg ∼1.9×10^−3^ µM) and rhSP-D (1 µg∼16.7×10^−3^ µM). SP-D has been implicated with both anti-inflammatory and pro-inflammatory roles. In the presence of LPS, SP-D induced pro-inflammatory NF-κB pathway in macrophages mediated by the interaction of its collagen domain with calreticulin/CD91complex [14]. *In vivo* and *in vitro* nitrosylation of the two cysteine residues at the N-terminus results in formation of S-nitrosothiol (SNO)-SP-D and disassembling of the oligomeric structure of SP-D. SNO-SP-D shows increased chemotaxis for macrophages and induced p38 phosphorylation and MAPK activation mediated by binding to calreticulin/CD91 complex [52]. Moreover, the MMP-9 cleaved SP-D (25 kDa) showed loss of innate immune functions including the agglutination and enhanced phagocytosis by macrophages, but retained LPS binding [33]. Thus, MMP-9 could potentially alter and regulate SP-D function during labor by cleaving the collagen domain [6], [33].

Observations from this study for occurrence of collectins in placenta are consistent with the earlier reports and suggest placental synthesis and differential expression of collectins during spontaneous labor. However, our observations merit further validation with a larger sample size and investigations on the molecular mechanisms regulating the differential expression of collectins at the feto-maternal interface.

**Figure 6:**
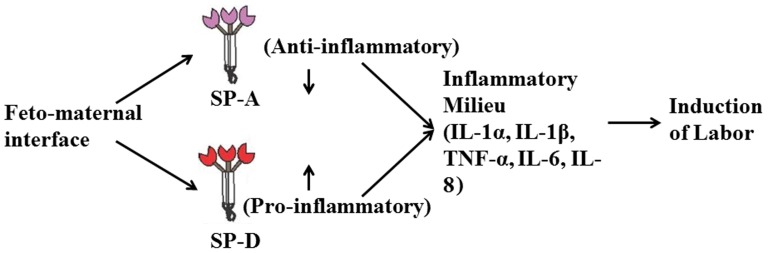
Schematic diagram of plausible functions of SP-A and SP-D at the term feto-maternal interface during labor.

In conclusion, the study emphasizes that SP-A and SP-D proteins associate with spontaneous labor and plausibly contribute to the anti-inflammatory and pro-inflammatory immune milieu of feto-maternal tissues respectively (**Fig. 6**).

## Supporting Information

Figure S1 **SP-A, SP-D and MBL transcripts in human term placenta.** The real time RT-PCR amplified products were resolved on 2% agarose gel electrophoresis; amplified transcripts SP-A2 (194 bps), SP-D (148 bps), MBL (224 bps) and 18S (171 bps).
(TIF)

Figure S2 **Analysis of placental SP-A, SP-D and MBL proteins.** A total of 30 µg term placental or 10–30 µg term human amniotic fluid (HAF) proteins were transferred onto the nitrocellulose membrane and probed with monoclonal antibody to a) SP-A or b) SP-D or c) MBL, while negative control was not probed with respective primary antibody (Ab control). The Marker lane shows molecular weight markers in kDa. # non-reducible SP-A dimer (68 kDa); * SP-A monomers in placenta.
(TIF)

Figure S3 **Purification of human SP-D from term amniotic fluid.** i) Silver stained gel of various fractions, 150 mM MnCl_2_ eluate (lane 2), 20 mM EDTA eluate (lane 3) and affinity column purified rhSP-D (lane 4); ii) Immunoblotting of various fractions using monoclonal antibody to SP-D, amniotic fluid (HAF; lane 1), term placenta (NP, lane 2), Mn^+2^ eluted nSP-D (lane 3) and affinity purified rhSP-D (lane 4).
(TIF)

Figure S4 **Cleavage of SP-D oligomers into 25 kDa fragments by MMP9 during spontaneous labor** (a) Western blot of term placental tissues using monoclonal antibody to SP-D (n = 3). (b) Densitometric analysis, bars are mean ±SEM, *p<0.05 (student t test). c) MTT assay was performed on the placental explants to test the effect of nSP-D and rhSP-D on their viability. Bars represent mean ±SEM.
(TIF)
