# Supplementary figures and images for: Differential Expression of Collectins in Human Placenta and Role in Inflammation during Spontaneous Labor
Source: PLoS One. 2014 Oct 10;9(10):e108815. doi: 10.1371/journal.pone.0108815 (PMC4193748; doi:10.1371/journal.pone.0108815)

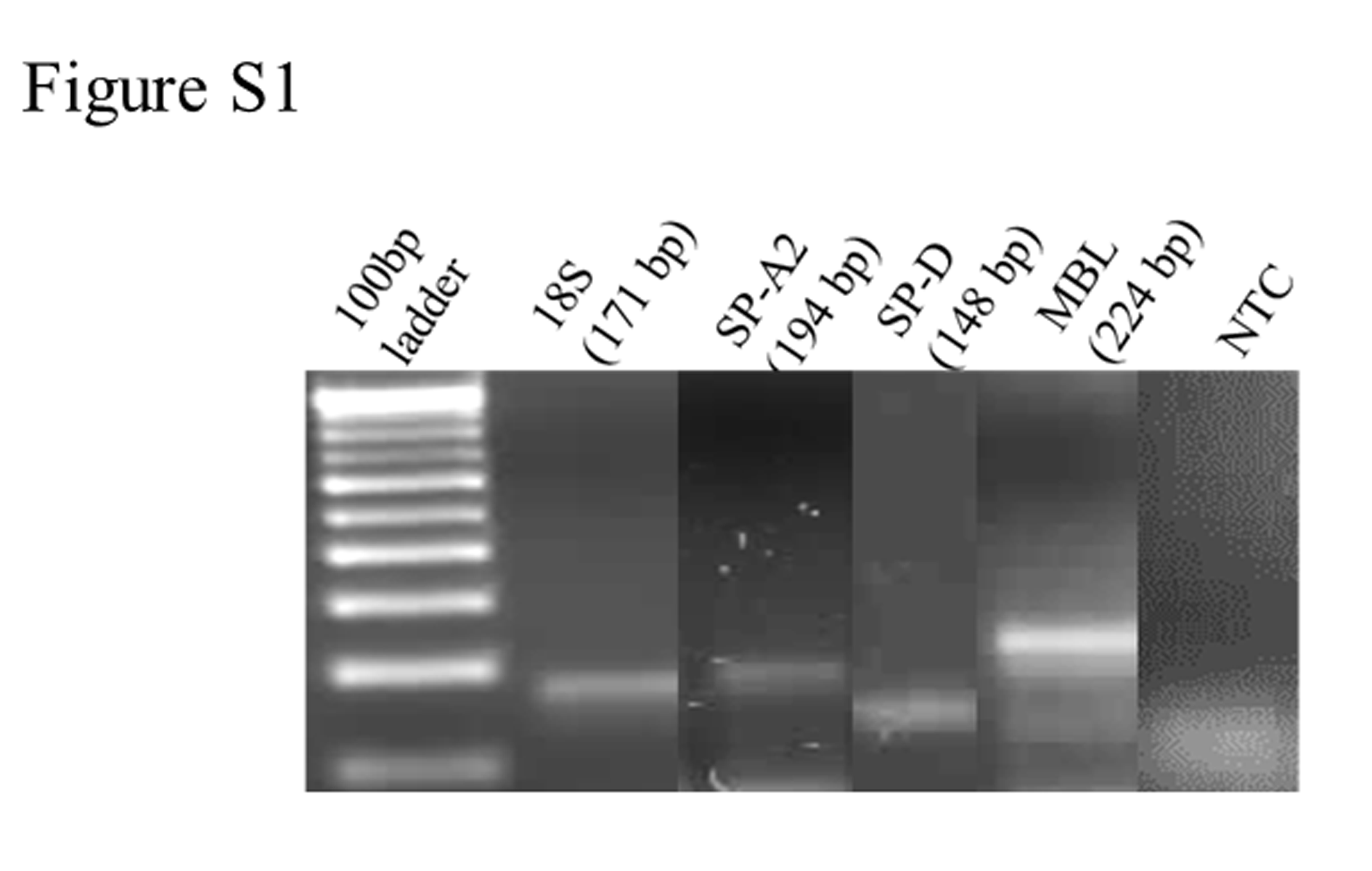

Supplement: Figure S1 — SP-A, SP-D and MBL transcripts in human term placenta. The real time RT-PCR amplified products were resolved on 2% agarose gel electrophoresis; amplified transcripts SP-A2 (194 bps), SP-D (148 bps), MBL (224 bps) and 18S (171 bps). (TIF) [file pone.0108815.s001.tif]

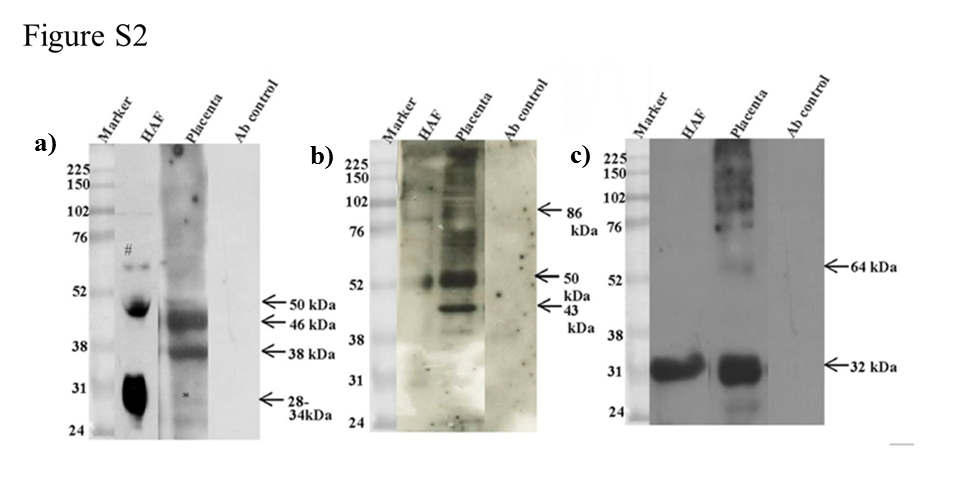

Supplement: Figure S2 — Analysis of placental SP-A, SP-D and MBL proteins. A total of 30 µg term placental or 10–30 µg term human amniotic fluid (HAF) proteins were transferred onto the nitrocellulose membrane and probed with monoclonal antibody to a) SP-A or b) SP-D or c) MBL, while negative control was not probed with respective primary antibody (Ab control). The Marker lane shows molecular weight markers in kDa. # non-reducible SP-A dimer (68 kDa); * SP-A monomers in placenta. (TIF) [file pone.0108815.s002.tif]

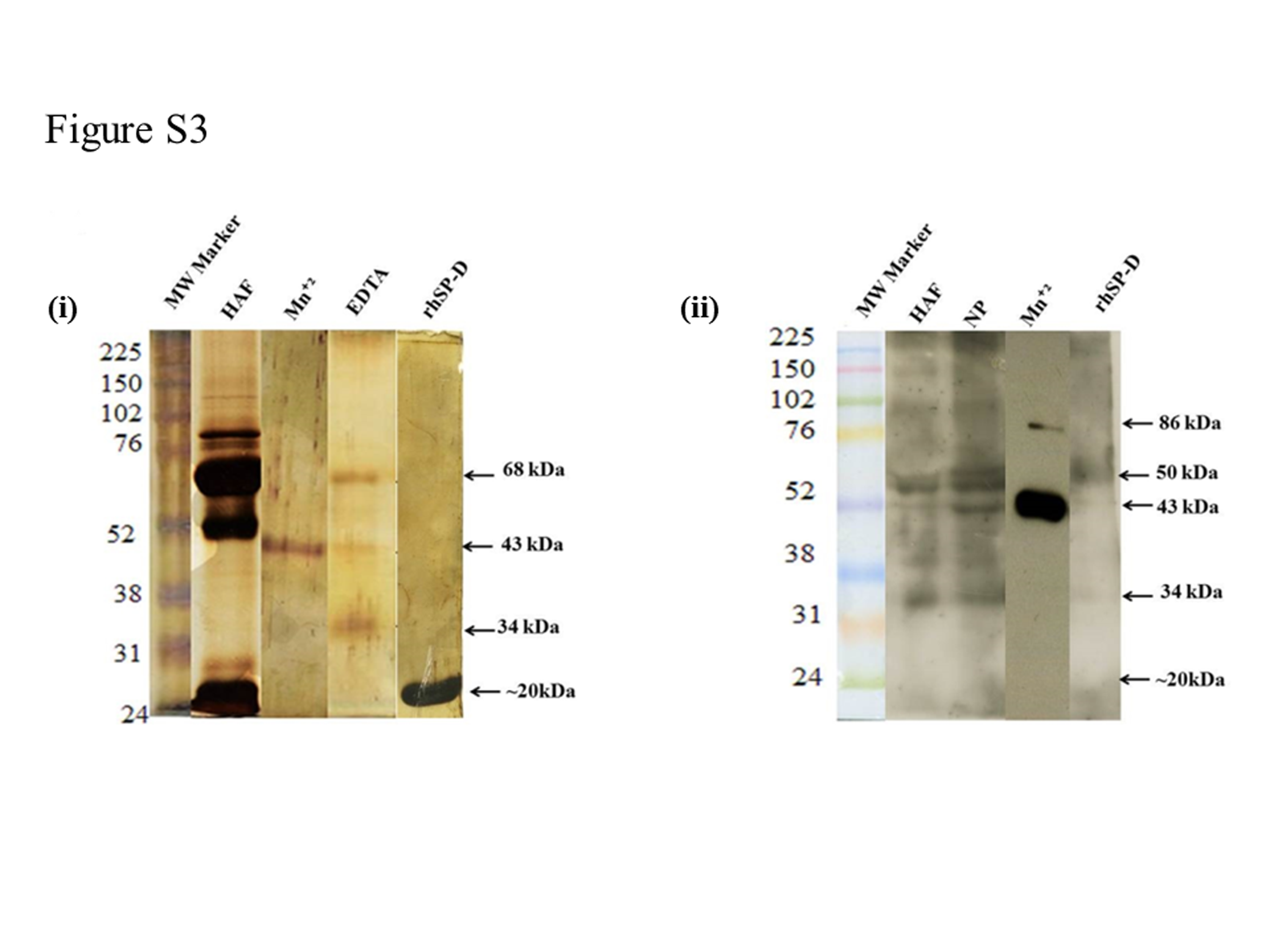

Supplement: Figure S3 — Purification of human SP-D from term amniotic fluid. i) Silver stained gel of various fractions, 150 mM MnCl2 eluate (lane 2), 20 mM EDTA eluate (lane 3) and affinity column purified rhSP-D (lane 4); ii) Immunoblotting of various fractions using monoclonal antibody to SP-D, amniotic fluid (HAF; lane 1), term placenta (NP, lane 2), Mn+2 eluted nSP-D (lane 3) and affinity purified rhSP-D (lane 4). (TIF) [file pone.0108815.s003.tif]

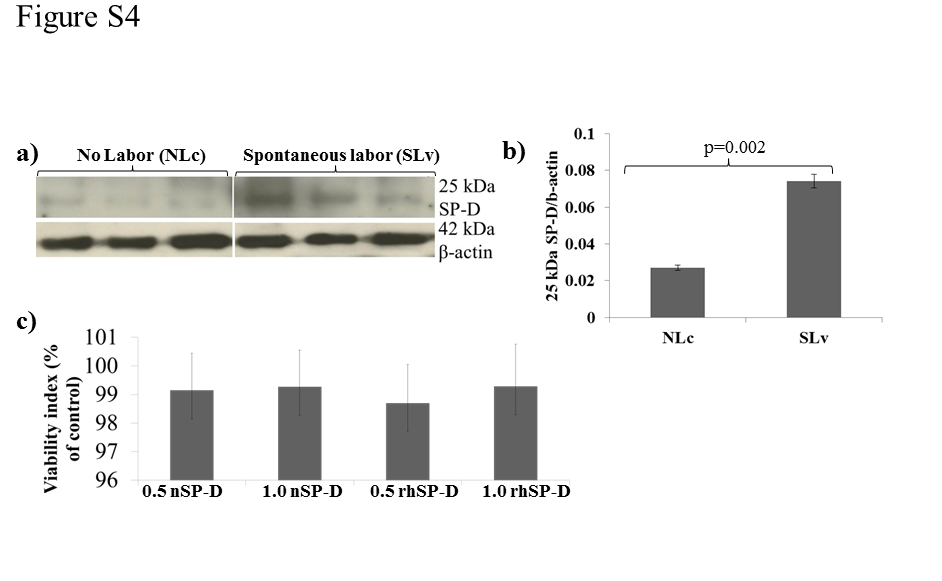

Supplement: Figure S4 — Cleavage of SP-D oligomers into 25 kDa fragments by MMP9 during spontaneous labor (a) Western blot of term placental tissues using monoclonal antibody to SP-D (n = 3). (b) Densitometric analysis, bars are mean ±SEM, *p<0.05 (student t test). c) MTT assay was performed on the placental explants to test the effect of nSP-D and rhSP-D on their viability. Bars represent mean ±SEM. (TIF) [file pone.0108815.s004.tif]
